# Belantamab mafodotin, bortezomib, and dexamethasone for RRMM in the Japan expansion cohort of the phase 3 DREAMM-7 trial

**DOI:** 10.1007/s12185-026-04211-4

**Published:** 2026-04-15

**Authors:** Tomoaki Fujisaki, Kohmei Kubo, Yasushi Hiramatsu, Ryosuke Ogawa, Taeko Yonekawa, Akira Endo, Hirofumi Nakano, Joe Lee, Lydia Eccersley, Hena Baig, Eric Lewis, Taku Fujii

**Affiliations:** 1 Matsuyama Red Cross Hospital, Ehime, Japan; 2 Aomori Prefectural Central Hospital, Aomori, Japan; 3 Japanese Red Cross Society Himeji Hospital, Hyogo, Japan; 4 JCHO Kyushu Hospital, Fukuoka, Japan; 5 GSK, Tokyo, Japan; 6 GSK, London, UK; 7 GSK, Ontario, Mississauga Canada; 8 GSK, Durham, NC USA

**Keywords:** Belantamab mafodotin, Multiple myeloma, Anti–b-cell maturation antigen, Monoclonal antibodies, Daratumumab

## Abstract

The randomized, phase 3 DREAMM-7 trial (NCT04246047) previously demonstrated the efficacy and safety of belantamab mafodotin, bortezomib, and dexamethasone (BVd) versus daratumumab, bortezomib, and dexamethasone (DVd) in patients with relapsed/refractory multiple myeloma (RRMM) and ≥ 1 prior therapy. The results in the Japan expansion cohort of DREAMM-7, consisting of 24 patients randomized to receive BVd (N = 10) or DVd (N = 14), are presented here. The median follow-up was 19.4 months (range, 1.3–30.3). Median progression-free survival (PFS) was not reached (NR; 95% CI, 7.0–NR) with BVd versus 11.1 months (95% CI, 4.9–NR) with DVd (PFS hazard ratio, 0.40; 95% CI, 0.11–1.52). The overall response rate was 90.0% (95% CI, 55.5–99.7) versus 71.4% (95% CI, 41.9–91.6); median duration of response was NR (95% CI, 9.7–NR) versus 14.5 months (95% CI, 3.5–NR). Safety trends in the Japan expansion cohort were similar to those in the global cohort. Ocular adverse reactions were more common with BVd and were manageable with dose modification. No new safety signals were reported. As in the global cohort, results in the Japan expansion cohort demonstrated the safety and efficacy of BVd in patients with RRMM and ≥ 1 prior therapy.

**Supplementary Information:**

The online version contains supplementary material available at https://doi.org/10.1007/s12185-026-04211-4.

## Introduction

In recent years, the development of novel therapies such as proteasome inhibitors, immunomodulators, and anti-CD38 monoclonal antibodies has evolved the treatment of multiple myeloma (MM) and improved patient outcomes [1–3]. For many patients, however, the disease becomes refractory to these treatments [4]. Indeed, the burden of relapsed/refractory multiple myeloma (RRMM) is significant, underscoring the need for effective combinations in the second line and beyond that incorporate new medicines with novel mechanism of action [5, 6].

B-cell maturation antigen (BCMA) is a receptor overexpressed on malignant plasma cells in MM and an established therapeutic target [7]. Belantamab mafodotin is a humanized, afucosylated, BCMA-targeting monoclonal antibody conjugated to the microtubule inhibitor monomethyl auristatin-F, by a protease-resistant cysteine linker [8, 9]. Belantamab mafodotin has diverse mechanisms of antitumor activity and has demonstrated efficacy and safety in the treatment of MM in a number of clinical trials as well as in real-world studies [10, 11]. The positive results from clinical trials have led to the approval of belantamab mafodotin combinations for RRMM in Japan [12].

The global, randomized, phase 3 DREAMM-7 trial (NCT04246047) evaluated belantamab mafodotin, bortezomib, and dexamethasone (BVd) versus daratumumab, bortezomib, and dexamethasone (DVd) in patients with RRMM and ≥ 1 prior line of therapy [13]. The trial met its primary endpoint of progression-free survival (PFS); BVd treatment conferred a statistically significant and clinically meaningful PFS benefit compared with DVd in patients with RRMM [13]. Additionally, a subsequent planned interim analysis of DREAMM-7 with a longer follow-up of approximately 3.3 years demonstrated a statistically significant and clinically meaningful improvement in overall survival with BVd compared with DVd [14]. Adverse events (AEs) with BVd were consistent with those previously described with belantamab mafodotin [13]. Ocular events, which are a known risk with belantamab mafodotin, were manageable with dose modifications and generally reversible [13].

In 2023, there were approximately 7000 new cases of MM and 4000 MM-related deaths in Japan [15]. The use of first-line lenalidomide and/or daratumumab appears to be increasing in Japanese patients with MM, which may lead to their disease becoming refractory to these agents [3]. As such, fewer regimens are available to patients who experience relapse, giving rise to a need for new therapeutic strategies.

While global studies have reported the benefit of BVd treatment in patients with RRMM, data specific to Japanese patients are important to ensure these findings are generalizable to the Japanese population. In this report, we present the results in the Japanese expansion cohort of DREAMM-7.

A plain language summary of this article is available in Supplementary Sect. “Introduction”.

## Methods

### Study design

The Japanese expansion cohort study of the randomized, phase 3 DREAMM-7 trial was initiated in August 2021. A list of participating sites are available in Supplementary Sect. “Methods”. Patient enrollment has been completed, and follow-up is ongoing. The analyses in this report are based on a data cutoff of 03 April 2024. The methodology of the DREAMM-7 study has been previously reported in detail (Figure S1) [13].

### Study participants

The study enrolled adults with RRMM who were treated with at least 1 prior line of therapy and had documented disease progression during or after the most recent therapy. Further details of the patient eligibility criteria are listed in the Supplementary Information, Sect. “Results”.

Briefly, patients were eligible if they had Eastern Cooperative Oncology Group performance status ≤ 2 and confirmed diagnosis of MM per International Myeloma Working Group (IMWG) criteria [16]. Exclusion criteria included: intolerance to daratumumab, refractoriness to daratumumab or any other anti-CD38 therapy, intolerance to bortezomib or refractoriness to bortezomib on a twice-weekly 1.3 mg/m^2^ schedule, or prior treatment with anti-BCMA therapy.

### Treatment

Patients were randomized in a 1:1 ratio to receive either BVd or DVd. Stratification factors included number of prior lines of therapy (1 vs 2/3 vs ≥ 4), prior bortezomib (yes vs no) and revised International Staging System stage (I vs II/III) at screening.

Both treatment groups received subcutaneous bortezomib (1.3 mg/m^2^ of body surface area on days 1, 4, 8, and 11 in 21-day cycles) and oral or intravenous dexamethasone (20 mg on the day of and the day after bortezomib) for the first eight cycles. The BVd group received intravenous belantamab mafodotin 2.5 mg/kg intravenously every 3 weeks while the DVd group received intravenous daratumumab 16 mg/kg once weekly in Cycles 1 to 3, every 3 weeks in Cycles 4 through 8, and every 4 weeks in Cycle 9 and beyond. The belantamab mafodotin dose could be reduced to 1.9 mg/kg or delayed to manage AEs and ophthalmic examination findings graded by the Keratopathy Visual Acuity (KVA) scale. Treatment was continued until the occurrence of disease progression, unacceptable toxic effects, withdrawal of consent, loss to follow-up, or death, whichever occurred first.

### Endpoints and assessments

The primary endpoint was PFS, defined as the time from randomization to the occurrence of confirmed disease progression or death. Disease progression was assessed by an independent review committee based on IMWG criteria.

The key secondary endpoints were: duration of response (DOR), defined as the time from first documented evidence of partial response (PR) or better until progressive disease or death due to any cause; overall survival, defined as the time from randomization to the date of death due to any cause; and minimal residual disease (MRD) negativity rate, defined as the percentage of patients who achieve MRD-negative status at least once during the period of confirmed complete response (CR) or better. MRD status was assessed by targeted next-generation sequencing at a 10^–5^ sensitivity threshold.

Supportive secondary endpoints included the following: overall response rate (ORR), defined as the percentage of patients with a confirmed PR or better as the best overall response (BOR); CR rate (CRR), defined as the percentage of patients with a confirmed complete response or better as the BOR; time to response, defined as the time (in months) between the date of randomization and the first documented evidence of response (PR or better) in patients who achieve a response; sustained MRD negativity rate, defined as the percentage of patients with MRD negativity confirmed by next-generation sequencing at least ≥ 1 year apart; PFS2, defined as the time from randomization to disease progression after initiation of a new anti-myeloma therapy or death from any cause, whichever occurred earlier. Responses were based on independent review committee assessment per IMWG criteria.

Health-related quality-of-life (QoL) assessments, including the European Organisation For Research and Treatment Of Cancer QoL Questionnaire 30-item Core module (EORTC QLQ-C30) and the EORTC QLQ 20-item Multiple Myeloma module (EORTC QLQ-MY20), were administered to patients based on the availability of the translated versions.

In a post-hoc analysis, PFS and response rates were evaluated in patients harboring high-risk cytogenetics, defined as t(4;14), t(14;16), 17p13del, or amp1q. Amp1q was not included in the definition of high-risk cytogenetics in baseline characteristics as these were based on the Revised International Staging System criteria used for stratification.

All AEs and serious AEs (SAEs) were collected from the start of treatment until at least 70 days following discontinuation of study treatment. AEs of special interest (AESIs) with belantamab mafodotin were ocular events, thrombocytopenic events, and infusion-related reactions. AESIs were based on a hybrid of terms identified in the electronic case report form. Ocular events consisted of ocular adverse reactions (graded using the Common Terminology Criteria for Adverse Events [CTCAE], version 5.0), ophthalmic examination findings from corneal examinations, and visual acuity changes (graded by the KVA scale). Patients receiving BVd underwent ophthalmic examination at screening, every 3 weeks prior to treatment administration up to at least the sixth dose of belantamab mafodotin, and then every 3 months if there were no ocular findings. Patients receiving DVd underwent ophthalmic examination at screening, at Cycle 6, and then every 6 months. More details on ophthalmic assessments are included in the Supplementary Information, Sect. “Discussion”. Severity of thrombocytopenic and infusion-related reaction AESIs were graded using the CTCAE. All AEs, SAEs, and AESIs were followed until the event was resolved, stabilized, or otherwise explained, or the patient was lost to follow-up.

### Statistical analysis

The efficacy analyses were based on intent-to-treat in the Japan expansion cohort, which included all randomized participants regardless of whether or not they received study treatment. The distribution of PFS was estimated using the Kaplan–Meier method; 95% confidence intervals (CIs) for the median, first and third quartiles were calculated with the Brookmeyer–Crowley method. The hazard ratio (HR) for PFS with its 95% CI was estimated using an unstratified Cox proportional-hazards model with treatment arm only as the explanatory variable. The distribution of DOR was estimated using the Kaplan–Meier method. For ORR based on the BOR and MRD negativity rate, the corresponding exact 95% CI was provided for each treatment arm in the Japanese cohort. The safety analyses were based on the safety population, defined as all randomized patients who received at least one dose of study treatment. The Japanese expansion cohort analyses were conducted after 11 PFS events had been observed. Due to the small sample size, statistical testing was not conducted on the differences in characteristics, efficacy, and safety between the BVd and DVd groups. As such, all analyses in the results are descriptive.

### Ethics

The trial was conducted in accordance with the Declaration of Helsinki and Good Clinical Practice guidelines. All patients provided written informed consent. The independent ethics committee or institutional review board at each site approved the protocols.

## Results

### Patient disposition

A total of 24 patients with RRMM in the Japan expansion cohort were randomized to either BVd (N = 10) or DVd (N = 14). At the data cutoff, 9 patients (4 in the BVd group vs 5 in the DVd group) were on study treatment, and 9 patients (5 in the BVd group vs 4 in the DVd group) were in follow-up (Table S1). Two patients withdrew from the study (1 in each group), with the reasons being withdrawal by patient due to burden of procedure in the BVd group and loss to follow-up in the DVd group. There were no deaths in the BVd group and four deaths in the DVd group.

Demographic and clinical characteristics were generally balanced between the BVd and DVd groups (Table 1). The mean age in the BVd and DVd groups was 67.9 and 70.9 years, respectively. The proportion of patients who had received one previous line of therapy was 60% (6/10) in the BVd group and 50% (7/14) in the DVd group. Baseline disease characteristics were generally similar between treatment groups except for the following: lytic bone lesions were more common in the BVd group than the DVd group (70% [7/10] vs. 29% [4/14]) and refractory disease was more common in the DVd group than the BVd group (64% [9/14] vs. 20% [2/10]). In the BVd group, 30% of patients (3/10) had disease refractory to lenalidomide, compared with 50% (7/14) in the DVd group.

**Table 1:** Patient demographics and clinical characteristics

	BVd (N = 10)	DVd (N = 14)	Total (N = 24)
Age, mean, years (SD)	67.9 (11.02)	70.9 (6.21)	69.6 (8.46)
ECOG performance status, n (%)*			
0	5 (50)	7 (50)	12 (50)
1	3 (30)	7 (50)	10 (42)
2	2 (20)	0	2 (8)
*R-ISS stage at screening, n (%)*			
I	4 (40)	4 (29)	8 (33)
II	6 (60)	10 (71)	16 (67)
III	0	0	0
Unknown	0	0	0
*Relapsed or refractory disease, n (%)*^*†*^			
Relapsed^‡^	7 (70)	5 (36)	12 (50)
Refractory^§^	2 (20)	9 (64)	11 (46)
Unknown	1 (10)	0	1 (4)
Cytogenetic risk, n (%)^¶^			
Standard	7 (70)	8 (57)	15 (63)
High	3 (30)	6 (43)	9 (38)
t(4;14)	3 (30)	5 (36)	8 (33)
t(14;16)	0	0	0
del(17p13)	0	1 (7)	1 (4)
*Other cytogenetic abnormalities, n (%)*			
del(13)	0	2 (14)	2 (8)
del(1p)	1 (10)	4 (29)	5 (21)
Hyperdiploidy	0	1 (7)	1 (4)
t(11;14)	0	1 (7)	1 (4)
t(14;20)	0	0	0
amp1q	5 (50)	7 (50)	12 (50)
Other	0	0	0
Extramedullary disease, n (%)	0	2 (14)	2 (8)
Lytic bone lesions, n (%)	7 (70)	4 (29)	11 (46)
Myeloma IgG, n (%)	6 (60)	11 (79)	17 (71)
*Previous lines of therapy, n (%)*			
1	6 (60)	7 (50)	13 (54)
2 or 3	3 (30)	5 (35)	8 (34)
≥ 4	1 (10)	2 (14)	3 (12)
*Time to relapse after most recent therapy, n (%)*			
≤ 12 months	0	4 (29)	4 (17)
> 12 months	10 (100)	10 (71)	20 (83)
*Previous treatments, n (%)*			
Bortezomib	9 (90)	13 (93)	22 (92)
Lenalidomide	8 (80)	12 (86)	20 (83)
Daratumumab	1 (10)	0	1 (4)
Autologous stem cell transplant	8 (80)	8 (57)	16 (67)
Lenalidomide refractory, n (%)	3 (30)	7 (50)	10 (42)

^*^ECOG performance status ranges from 0 (fully active, no restrictions) to 5 (death). ^†^Categorized per IMWG based on investigator assessment. ^‡^Collected in the CRF as follows: PD after > 60 days of stopping treatment (includes eCRF categories: relapsed < 6 months, 6 to 12 months, and > 12 months). ^§^Collected in the CRF as follows: PD on treatment or within 60 days of stopping treatment (includes eCRF categories: relapsed < 60 days, refractory, primary refractory, relapsed, and refractory). ^¶^Standard cytogenetic risk was defined by negative results for all high-risk abnormalities: t(4;14), t(14;16), and del(17p13). High cytogenetic risk was defined by the presence of at least one high-risk abnormality: t(4;14), t(14;16), or del(17p13). High-risk abnormalities were assessed by interphase fluorescence in situ hybridization with the following central laboratory thresholds

BVd, belantamab mafodotin, bortezomib, and dexamethasone; CRF, case report form; DVd, daratumumab, bortezomib, and dexamethasone; ECOG, Eastern Cooperative Oncology Group; eCRF, electronic case report form; IMWG, International Myeloma Working Group; IgG, immunoglobulin G; PD, progressive disease; R-ISS, Revised International Staging System; SD, standard deviation

### Efficacy

**Fig. 1:**
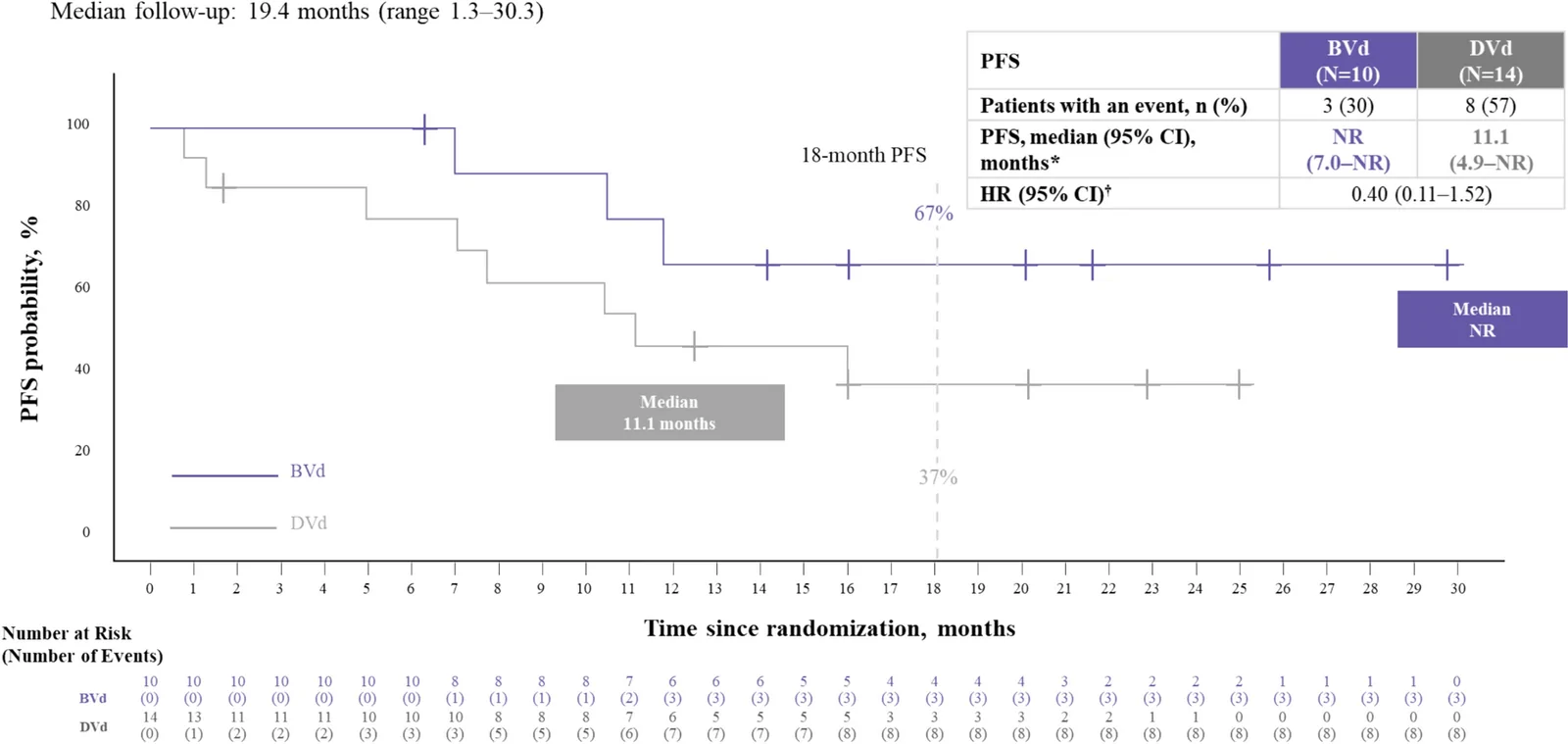
Kaplan–Meier curve of PFS. *95% CIs were estimated using the Brookmeyer-Crowley method. ^†^HR was estimated using an unstratified Cox proportional hazards model with treatment arm as the sole explanatory variable. BVd, belantamab mafodotin, bortezomib, and dexamethasone; CI, confidence interval; DVd, daratumumab, bortezomib, and dexamethasone; HR, hazard ratio; NR, not reached; PFS, progression-free survival

The median duration of follow-up was 19.4 months (range, 1.25 to 30.32). The median PFS was not reached (NR; 95% CI, 7.0 to NR) in the BVd group and 11.1 months (95% CI, 4.9 to NR) in the DVd group (HR, 0.40; 95% CI, 0.11–1.52). There were three PFS events (30%) in the BVd group, all of which were disease progression, and eight events (57%) in the DVd group, seven of which were disease progression and one was death (Fig. 1). PFS rate at 18 months was higher in the BVd group than the DVd group (67% vs. 37%). At the time of data cutoff, there were no deaths in the BVd group and four deaths in the DVd group. Of the four deaths in the DVd group, three were attributed to disease progression, and one to pneumonitis.

Treatment response and MRD outcomes are reported in Table 2. ORR was 90.0% (9/10; 95% CI, 55.5–99.7) in the BVd group and 71.4% (10/14; 95% CI, 41.9–91.6) in the DVd group. CRR was also higher in the BVd group (40.0% [4/10]; 95% CI, 12.2–73.8) than in the DVd group (14.3% [2/14]; 95% CI, 1.8–42.8). Median DOR was NR (95% CI, 9.7 to NR) in the BVd group and 14.5 months (95% CI, 3.5 to NR) in the DVd group. The median time to response was short and similar between treatment groups: 0.82 months (range, 0.76–3.58) in the BVd group and 1.13 months (range, 0.72–1.51) in the DVd group. The proportion of patients who achieved MRD negativity plus CR or better was 20.0% (2/10; 95% CI, 2.5–55.6) in the BVd group and 14.3% (2/14; 95% CI, 1.8–42.8) in the DVd group. A swimmer plot of belantamab mafodotin treatment duration and response is illustrated in Figure S2. No patient had MRD negativity plus CR or better of ≥ 12 months at the time of data cutoff; however, this is in the context of a median follow-up of 19.4 months.

**Table 2:** Treatment response and MRD

	BVd (N = 10)	DVd (N = 14)
*Best overall response, n (%)*		
Stringent complete response	2 (20.0)	1 (7.1)
Complete response	2 (20.0)	1 (7.1)
Very good partial response	4 (40.0)	6 (42.9)
Partial response	1 (10.0)	2 (14.3)
Minimal response	1 (10.0)	0
Stable disease	0	3 (21.4)
Progressive disease	0	1 (7.1)
Not evaluable	0	0
Overall response, n (%)*	9 (90.0)	10 (71.4)
95% CI	(55.5–99.7)	(41.9–91.6)
Complete response or better, n (%)	4 (40.0)	2 (14.3)
95% CI	(12.2–73.8)	(1.8–42.8)
Response duration, median, months (95% CI)^†^	NR (9.7–NR)	14.5 (3.5–NR)
Time to first response, median, months (range)^‡^	0.82 (0.76–3.58)	1.13 (0.72–1.51)
MRD-negative status, n (%)^§^	2 (20.0)	2 (14.3)
95% CI	(2.5–55.6)	(1.8–42.8)
MRD-negative status sustained for ≥ 12 months, n (%)^§^	0	0
95% CI	(0.0–30.8)	(0.0–23.2)

^*^Patients achieving a confirmed partial response or better. ^†^Duration of response is defined as the time from the first documented evidence of a partial response or better to the occurrence of disease progression or death from any cause. ^‡^Time to first response is defined as the time from the date of randomization to the first documented evidence of a partial response or better among patients who had a confirmed partial response or better. ^§^MRD-negative status for patients with complete response or better, assessed by means of next-generation sequencing at a 10^−5^ sensitivity threshold

BVd, belantamab mafodotin, bortezomib, and dexamethasone; CI, confidence interval; DVd, daratumumab, bortezomib, and dexamethasone; CI, confidence interval; DVd, daratumumab, bortezomib, and dexamethasone; MRD, minimal residual disease; NR, not reached.

The proportion of patients with ≥ 1 high-risk cytogenetic abnormality, defined as t(4;14), t(14;16),17p13del or amp1q, was 70% (7/10) in the BVd group and 71% (10/14) in the DVd group. Among these patients, 43% (3/7) in the BVd group and 50% (5/10) in the DVd group experienced a PFS event (Table S2). PR or better was achieved by 86% (6/7) in the BVd group and 70% (7/10) in the DVd group; very good PR (VGPR) or better was achieved by 71% (5/7) in the BVd group and 50% (5/10) in the DVd group; CR or better was achieved by 29% (2/7) in the BVd group and 20% (2/10 in the DVd group).

Three patients in the BVd group and 8 in the DVd group experienced progression on treatment and received subsequent antimyeloma therapy (Table S3). The most common subsequent therapy after BVd was steroids, which all 3 patients received, followed by monoclonal antibodies and proteasome inhibitors, which 2 patients received. The most common subsequent therapies after DVd were steroids and immunomodulators, which all 8 patients received, followed by monoclonal antibodies, which 6 patients received. Median PFS2 was NR in the BVd group and 17.5 months in the DVd group (HR, 0.13; 95% CI, 0.02–1.06).

The mean EORTC QLQ-C30 score at baseline was 76.5 (range, 56.8–97.4) in the BVd group (n = 10) and 84.8 (range, 57.6–94.4) in the DVd group (n = 14). At the end of treatment, the mean EORTC QLQ-C30 score was 61.1 (range, 44.7–73.5) in the BVd group (n = 5) and 82.3 (range, 49.4–97.4) in the DVd group (n = 9). The mean change in EORTC QLQ-C30 score from baseline to the end of treatment was -8.9 and -0.2 in the BVd and DVd groups, respectively.

The mean EORTC QLQ-MY20 score at baseline was 28.3 (range, 0.0–66.7) in the BVd group (n = 10) and 11.5 (range, 0.0–44.4) in the DVd group (n = 14). At the end of treatment, the mean EORTC QLQ-MY20 score was 36.7 (range, 11.1–50.0) in the BVd group (n = 5) and 13.0 (range, 0.0–33.3) in the DVd group (n = 9). The mean change in EORTC QLQ-MY20 score from baseline to the end of treatment was 0.0 and − 1.9 in the BVd and DVd groups, respectively.

### Safety

An overview of AEs reported in the study is presented in Table 3. All patients in the BVd and DVd groups had at least one AE. An AE leading to discontinuation of study treatment was reported in 1 patient (diarrhea) in the BVd group and no patients in the DVd group. The incidence of SAEs was higher in the BVd group (70% [7/10]) than the DVd group (14% [2/14]), while the incidence of treatment-related SAEs was similar between both groups (20% [2/10] vs. 14% [2/14]). None of the SAEs in the BVd group were fatal; one fatal treatment-related SAE (pneumonitis) was reported in the DVd group.

**Table 3:** Safety profile of BVd versus DVd

	BVd (N = 10)	DVd (N = 14)
Any AE, n (%)	10 (100)	14 (100)
AEs related to any study treatment*	10 (100)	14 (100)
Grade 3/4 AEs	10 (100)	12 (86)
Grade 3/4 AEs related to any study treatment*	10 (100)	12 (86)
AEs leading to permanent discontinuation of any study treatment	1 (10)	0
AEs related to any study treatment and leading to permanent discontinuation of any study treatment*	1 (10)	0
AEs leading to dose reduction	9 (90)	9 (64)
AEs leading to dose interruption/delay	9 (90)	12 (86)
Any SAE, n (%)	7 (70)	2 (14)
SAEs related to any study treatment*	2 (20)	2 (14)
Fatal SAEs	0	1 (7)
Fatal SAEs related to any study treatment*	0	1 (7)^†^

^*^ “Related to any study treatment” included responses of “Yes” and missing responses to the following question: “Is there a reasonable possibility that the AE may have been caused by the study treatment?”. Study treatments included dexamethasone, bortezomib, daratumumab, and belantamab mafodotin. ^†^The fatal treatment-related SAE in the DVd group was pneumonitis

AE, adverse event; BVd, belantamab mafodotin, bortezomib, and dexamethasone; DVd, daratumumab, bortezomib, and dexamethasone; SAE, serious adverse event

The incidence of infections was 60% (6/10) in the BVd group and 64% (9/14) in the DVd group. Seven serious infection AEs were reported in 4 patients in the BVd group and none in the DVd group. The serious infection AEs included one event each of eyelid infection, pneumonia, pneumococcal pneumonia, sepsis, and urinary tract infection, and two events of COVID-19. Both serious COVID-19 infections were grade 3 events. One occurred 367 days after the first dose of BVd, and was resolved in 43 days; the other occurred 110 days after the first dose of BVd, and was resolved in 19 days. Both events were considered not related to the treatment. The overall frequency of COVID-19 was similar between BVd and DVd (20% [2/10] and 14% [2/14], respectively).

Table S4 lists the treatment-emergent AEs (TEAEs) that occurred in ≥ 20% of patients in either treatment arm. All patients in the BVd and DVd groups experienced TEAEs. In the BVd and DVd groups, the most common TEAE was thrombocytopenia, which occurred in 70% (7/10) and 50% (7/14) of patients, respectively. Thrombocytopenic AESIs occurred in 100% (10/10) and 86% (12/14) of patients in the BVd and DVd groups, respectively, and infusion-related AESIs occurred in 10% (1/10) and 21% (3/14) of patients in the BVd and DVd groups, respectively (Table S5).

CTCAE-graded ocular adverse reactions occurred in 80% of patients (8/10) in the BVd group and 7% (1/14) in the DVd group (Table S5). In the BVd group, foreign body sensation in eyes and blurred vision were the most frequently reported ocular adverse reactions, each occurring in 70% of patients. No CTCAE grade ≥ 3 ocular adverse reactions were reported in either treatment group. In general, ocular adverse reactions in the BVd group were manageable with dose delays/interruptions. In 7 of 8 patients in the BVd group and 1 of 1 in the DVd group, the first occurrence of ocular adverse reaction resolved prior to the end of treatment exposure. Follow-up is ongoing for the patient in the BVd group with an unresolved event at data cutoff. All 10 patients in the BVd group had ophthalmic examination findings, as assessed by an investigator using the KVA scale (Table S6). Grade ≥ 3 events were reported in 80% of patients. Ophthalmic examination findings led to dose reduction in 40% of patients and dose interruptions/delays in 100%. There were 29 occurrences of dose delays for belantamab mafodotin, with a median duration of 81 days (range, 18–414). Dose delays for daratumumab were reported in 43% of patients (6/14) in the DVd group, with 11 occurrences and a median duration of 6 days. In the BVd group, the median relative dose intensities (RDIs) for belantamab mafodotin, bortezomib, and dexamethasone were 33.9% (range, 19.1–55.7), 53.4% (range, 22.5–88.4), and 82.0% (range, 36.8–96.9), respectively. In the DVd group, the median RDI for daratumumab was 100.0% (range, 57.6–104.0) in Cycles 1–3, 100.0% (range, 71.4–103.4) in Cycles 4–8, and 100.2% (range, 85.2–103.2) in Cycles 9 and beyond; the median RDIs for bortezomib and dexamethasone were 91.6% (range, 63.8–110.6) and 84.9% (range, 24.6–105.0), respectively. The first occurrence of ophthalmic examination findings resolved prior to the end of treatment exposure in all 10 patients in the BVd group. None of the patients discontinued treatment due to ocular events.

## Discussion

This analysis of the Japanese expansion cohort of DREAMM-7 showed that BVd demonstrated favorable efficacy compared with DVd and a manageable safety profile in Japanese patients with RRMM and at least one prior therapy.

Disease characteristics in the Japan expansion cohort were comparable to those in the global cohort [13]. The observed efficacy of BVd in the Japan cohort is aligned with that in the global study [13]. In the global cohort, the BVd group demonstrated a longer median PFS than the DVd group (36.6 vs. 13.4 months; HR for PFS, 0.41; P < 0.001) and a greater ORR, CRR, and DOR [13]. The proportion of patients who achieved MRD negativity plus CR or better was also higher in the BVd group than the DVd group in the global cohort [13]. Ocular events observed in the BVd group in the global cohort were manageable with dose modifications. In the Japan expansion cohort, the BVd group demonstrated longer PFS than the DVd group at the data cutoff, with an HR of 0.40 (95% CI, 0.11–1.52). Similar to the primary analysis, ORR, CRR, and DOR were greater with BVd than DVd. The proportion of patients who achieved CR or better and MRD negativity, which is associated with improved survival, was higher in the BVd group than the DVd group [17]. Similar trends for treatment response were observed in patients with ≥ 1 high-risk cytogenetic abnormality. There was no substantial difference in the change in patient-reported QoL from baseline to end of treatment between both groups, similar to the global cohort [13].

Overall, the findings from the Japan expansion cohort suggest that BVd is effective in Japanese patients with RRMM. With the increasing use of lenalidomide in Japanese patients in the first-line setting, more patients are likely to have lenalidomide-refractory disease, resulting in poor outcomes with retreatment at subsequent relapse [3]. In the BVd arm of the Japan expansion cohort, 30% of patients had lenalidomide-refractory disease. Real-world data have shown that patients with lenalidomide-refractory disease had poorer survival outcomes than those with non-refractory disease regardless of whether lenalidomide was used in subsequent treatments [18]. Despite this, the benefit of BVd as a second-line treatment and beyond was demonstrated in this study. This is in line with findings from a subgroup analysis of patients with lenalidomide-refractory disease in the global cohort, with a longer median PFS observed in the BVd group (25.0 months; 95% CI, 18.1–NR) than the DVd group (8.6 months; 95% CI, 6.4–13.5) [19]. Thus, BVd is a potential solution to address the current unmet need in the second line and beyond.

Safety trends observed in the Japan expansion cohort are also aligned with those in the global study [13]. The incidence of any AE in the BVd group in the Japan expansion cohort was consistent with the safety profile in the global cohort. While the incidence of SAEs was slightly higher in the Japan cohort (70%) than the global cohort (50%), none of the SAEs were fatal in the Japan cohort, and 10% of patients in the global cohort experienced fatal SAEs [13]. The incidence of infections in the BVd and DVd groups in the Japan cohort were similar to those in the global cohort [13]. CTCAE-graded ocular adverse reactions were more common in the BVd group than the DVd group (80% vs 7%), similar to in the global cohort analysis (79% vs. 29%) [13]. Ophthalmic examination findings graded by the KVA scale were also more common in the BVd group than the DVd group in both cohorts. In general, ocular adverse reactions and ophthalmic examination findings in the BVd group were manageable with dose modifications (dose delays/interruptions and reductions). None of the patients discontinued treatment due to ocular events in the Japan cohort. A post-hoc analysis of the global cohort found that dose modifications were useful in managing ocular AEs while maintaining treatment benefit of BVd, further demonstrating that the risk of ocular events associated with belantamab mafodotin is manageable [20]. Regular ophthalmic examinations remain a critical component of managing ocular AEs associated with belantamab mafodotin, and can be enhanced by close collaboration with ophthalmologists even before initiating treatment. Thrombocytopenic AESIs were slightly higher in the BVd and DVd groups in the Japan cohort (100 and 86%, respectively) than the global cohort (87 and 65%, respectively), although none led to discontinuation of study treatment. No new safety signals were observed.

The RDI of belantamab mafodotin was lower in the Japan expansion cohort than that reported in the global cohort (33.9% vs. 51%) [13]. This difference may be at least partly explained by a higher proportion of patients who experienced belantamab mafodotin dose delay in the Japan cohort compared with the global cohort (100% vs. 88%), and a longer median duration of dose delay (81 days vs. 54 days) [21]. This may have contributed to the lower RDI observed in Japanese patients. Although all Japanese patients experienced grade ≥ 2 ocular events based on the KVA scale, which requires dose modification, it should be noted that the proportion of patients with grade ≥ 3 ocular events based on the KVA scale, which are a driver of dose adjustments, were similar in the Japan and global cohorts (80 and 74%, respectively) [13]. In addition, the number of Japanese patients included in this analysis was limited, making it difficult to definitively determine the underlying reasons for the observed difference in RDI. Importantly, the efficacy and safety profiles of belantamab mafodotin in combination with bortezomib and dexamethasone in the Japan cohort were generally consistent with those observed in the global population, and the lower RDI did not appear to compromise the overall benefit–risk profile in this cohort.

A limitation of this study is the small sample size of 24 patients, which may impact the generalizability of the findings to the wider Japanese population. Because belantamab mafodotin has known ocular toxic effects, another potential limitation is the reporting bias of ocular events toward the BVd group due to the higher frequency of ocular examinations. Additionally, the open-label design of the study could lead to bias in investigators’ interpretations.

In Japanese patients with RRMM and at least one 1 prior therapy, BVd showed favorable efficacy compared with DVd and had a manageable safety profile. No new safety concerns were seen in Japanese patients. Overall findings were consistent with those in the global DREAMM-7 cohort. The results from the Japan expansion cohort demonstrate that BVd, with a novel mechanism of action, could be a promising treatment option for the increasing number of patients who have had one or more prior therapies.

## Supplementary Information

Below is the link to the electronic supplementary material.Supplementary file1 (DOCX 659 KB)

## Data Availability

Please refer to GSK weblink to access GSK’s data sharing policies and as applicable seek anonymized subject level data via the link https://www.gsk-studyregister.com/en/.
