# Supplementary material for: Belantamab mafodotin, bortezomib, and dexamethasone for RRMM in the Japan expansion cohort of the phase 3 DREAMM-7 trial
Source: Int J Hematol. 2026 Apr 15;124(3):370–9. doi: 10.1007/s12185-026-04211-4 (PMC13529901; doi:10.1007/s12185-026-04211-4)
Supplement: Supplementary file 1 [file 12185_2026_4211_MOESM1_ESM.docx]

**SUPPLEMENTARY INFORMATION**

**Supplementary Section 1: Plain language summary (254 words)**

**Why was this study done?**

Multiple myeloma is a cancer of plasma cells, a type of white blood cell. In the past few years, new drugs have been developed to stop this cancer from getting worse, helping patients live longer. However, for many patients, these drugs stop working over time, and the cancer comes back or worsens, also known as relapsed/refractory multiple myeloma.

Belantamab mafodotin, also known as belamaf, is a new drug that helps these patients. DREAMM-7 is a world-wide clinical study that looked at two combinations of drugs used in multiple myeloma: belamaf, bortezomib, and dexamethasone and the standard treatment of daratumumab, bortezomib, and dexamethasone, to find out which treatment works better in patients with relapsed/refractory multiple myeloma. The results showed that the belamaf combination was more effective at stopping the cancer from getting worse than the standard treatment.

**What did we do?**

We analyzed the results in Japanese patients in the DREAMM-7 study to see if the results were similar to those in the main group of patients.

**What did we find?**

Similar to the world-wide study, we found that Japanese patients who received treatment with belamaf, bortezomib, and dexamethasone lived longer without their cancer getting worse compared with patients who received standard treatment. The side effects of the treatment were also similar in Japanese patients compared to the world-wide study.

**What do these findings mean?**

Belamaf, bortezomib, and dexamethasone can be an effective option for treating relapsed/refractory multiple myeloma in Japanese patients who have been treated with other drugs before.

**Supplementary Section 2: Participating sites**

| Site name | City/ Prefecture | Principal investigator |
| --- | --- | --- |
| Ogaki Municipal Hospital | Gifu | Hiroshi Kosugi |
| Matsuyama Red Cross Hospital | Ehime | Tomoaki Fujisaki |
| Shibukawa Medical Center | Gunma | Morio Matsumoto |
| Okayama Medical Center | Okayama | Kazutaka Sunami |
| Nagoya City University Hospital | Aichi | Shinsuke Iida |
| Kobe City Medical Center General Hospital | Hyogo | Yuya Nagai |
| Hokkaido University Hospital | Hokkaido | Hideki Goto |
| Suwa Red Cross Hospital | Nagano | Yasushi Senoo |
| Fukushima Medical University Hospital | Fukushima | Takayuki Ikezoe |
| Toyohashi Municipal Hospital | Aichi | Shingo Kurahashi |
| University Hospital, Kyoto Prefectural University of Medicine | Kyoto | Junya Kuroda |
| Osaka Saiseikai Nakatsu Hospital | Osaka | Ryosuke Yamamura |
| NTT Medical Center Tokyo | Tokyo | Kensuke Usuki |
| Japanese Red Cross Society Himeji Hospital | Hyogo | Yasushi Hiramatsu |
| Shizuoka Cancer Center | Shizuoka | Takashi Ikeda |
| National Hospital Organization Kyushu Medical Center | Fukuoka | Hiromi Iwasaki |
| Osaka Metropolitan University Hospital | Osaka | Hirohisa Nakamae |
| Kameda General Hospital | Chiba | Kosei Matsue |
| Japan Community Health care Organization Kyushu Hospital | Fukuoka | Ryosuke Ogawa |
| Aomori Prefectural Central Hospital | Aomori | Kohmei Kubo |
| Anjo Kosei Hospital | Aichi | Masashi Sawa |
| University Hospital of Occupational and Environmental Health | Fukuoka | Junichi Tsukada |
| Shonan Kamakura General Hospital | Kanagawa | Shuku Sato |
| Yokohama Municipal Citizen’s Hospital | Kanagawa | Tomonori Nakazato |
| Japanese Red Cross Fukuoka Hospital | Fukuoka | Kazuki Tanimoto |
| Kochi Health Sciences Center | Kochi | Toshi Imai |

**Supplementary Section 3: DREAMM-7 Japanese expansion cohort selected patient eligibility criteria**

**Inclusion criteria**

Patients were eligible to be included if they met all the following criteria:

1. Capable of giving signed informed consent which includes compliance with the requirements and restrictions listed in the consent form.
2. Male or female, 18 years or older (at the time consent is obtained).
3. Confirmed diagnosis of MM as defined by the International Myeloma Working Group (IMWG) criteria.
4. Previously treated with at least 1 prior line of MM therapy, and must have documented disease progression during or after their most recent therapy (Note: induction + autologous stem cell transplant [ASCT] + maintenance is counted as 1 line of therapy).
5. Eastern Cooperative Oncology Group (ECOG) performance status ≤2.
6. Patients with a history of ASCT were eligible for study participation provided that the ASCT was >100 days prior to initiating study treatment, AND the patient did not have any active bacterial, viral, or fungal infections.
7. At least one aspect of measurable disease defined as:
   a. Urine M-protein excretion ≥200 mg per 24-hour, OR
   b. Serum M-protein concentration ≥0.5 g/dL, OR
   c. Serum free light chain (FLC) assay: involved FLC level ≥10 mg/dL (≥100 mg/liter) and an abnormal serum free light chain ratio (<0.26 or >1.65)
8. No prior treatment-related toxicities (defined by National Cancer Institute-Common Toxicity Criteria for Adverse Events [CTCAE, v5.0]) above Grade 1 at the time of enrollment, except for alopecia.
9. Adequate organ system functions as defined by laboratory assessments listed in the table below:

| Organ system and laboratory tests | Laboratory values |
| --- | --- |
| Hematologic |  |
| Absolute neutrophil count^a^ | ≥1.0 × 10^9^/L |
| Hemoglobin | ≥8.0 g/dL |
| Platelets | ≥75 × 10^9^/L |
| Hepatic |  |
| Total bilirubin | ≤1.5 × upper limits of normal (ULN)  (Isolated bilirubin >1.5 × ULN is acceptable if bilirubin is fractionated and direct bilirubin is <35%) |
| Alanine aminotransferase | ≤2.5 × ULN |
| Renal |  |
| eGFR^b^ | ≥30 mL/min/1.73 m^2^ |
| Urine dipstick for protein | Negative/trace [if ≥1+, only eligible if confirmed ≤500 mg/g (56 mg/mmol) by albumin/creatinine ratio (spot urine from first void)] |
| OR |  |
| Albumin/creatinine ratio (from spot urine) | ≤500 mg/g (56 mg/mmol) |

^a^Without growth factor support, blood transfusion or platelet stimulating agents for the past 14 days, excluding erythropoietin. ^b^As calculated by Modified Diet in Renal Disease formula.

10. Female patients:
    a. Contraceptive use by women should be consistent with local regulations regarding the methods of contraception for those participating in clinical studies.
    b. Female patients were eligible to participate if they were not pregnant or breastfeeding, and fulfilled at least one of the following criteria:
       i. Is not a woman of childbearing potential (WOCBP) OR
       ii. Is a WOCBP using a highly effective contraceptive method (with a failure rate of <1% per year) during the period of intervention and for 4 months after the last dose of belantamab mafodotin, 3 months from the last dose of daratumumab, and 7 months from the last dose of bortezomib, whichever is longer; WOCBP must have a negative highly sensitive serum pregnancy test within 72 hours of dosing on Cycle 1 Day 1.
11. Male participants:
    a. Contraceptive use by men should be consistent with local regulations regarding the methods of contraception for those participating in clinical studies.
    b. Male participants are eligible to participate if they agree to the following from the time of first dose of study until 6 months after the last dose of belantamab mafodotin, and 4 months from the last dose of bortezomib:
       i. Refrain from donating sperm AND
       ii. Be abstinent from heterosexual intercourse on a long-term and persistent basis and agree to remain abstinent OR agree to use a male condom (even if they have undergone a successful vasectomy) and female partner to use an additionally highly effective contraceptive method

**Exclusion criteria**

Patients were excluded from the study if any of the following criteria were met:

1. Intolerant to daratumumab.
2. Refractory to daratumumab or any other anti-CD38 therapy (defined as progressive disease during treatment with anti-CD38 therapy, or within 60 days of completing that treatment).
3. Intolerant to bortezomib, or refractory to bortezomib (defined as progressive disease during treatment with a bortezomib-containing regimen of 1.3 mg/m2 twice weekly, or within 60 days of completing that treatment). Note: participants with progressive disease during treatment with a weekly bortezomib regimen are allowed.
4. Ongoing Grade 2 or higher peripheral neuropathy or neuropathic pain.
5. Prior treatment with anti-BCMA therapy.
6. Prior treatment with a monoclonal antibody within 30 days of receiving the first dose of study drugs, or treatment with an investigational agent or approved systemic antimyeloma therapy (including systemic steroids) within 14 days or 5 half-lives of receiving the first dose of study drugs, whichever is shorter.
7. Plasmapheresis within 7 days prior to the first dose of study drug.
8. Prior radiotherapy to a large pelvic area. Bridging radiotherapy otherwise is allowed. Note: Disease assessment should be repeated if radiotherapy is done prior to first dose of study drug within screening window.
9. Prior allogenic stem cell transplant. Note: Participants who have undergone syngeneic transplant will be allowed, only if no history of graft-versus-host disease.
10. Any major surgery within 4 weeks prior to the first dose of study drug. Exception allowed for bone stabilizing surgery after consultation with medical monitor.
11. Presence of active renal condition (infection, requirement for dialysis or any other condition that could affect participant’s safety). Participants with isolated proteinuria resulting from MM are eligible, provided they have adequate organ system functions as defined by laboratory assessments.
12. Any serious and/or unstable pre-existing medical, psychiatric disorder or other conditions (including lab abnormalities) that could interfere with participant’s safety, obtaining informed consent or compliance to the study procedures.
13. Evidence of active mucosal or internal bleeding.
14. Cirrhosis or current unstable liver or biliary disease per investigator assessment defined by the presence of ascites, encephalopathy, coagulopathy, hypoalbuminemia, esophageal or gastric varices, persistent jaundice. Note: Stable non-cirrhotic chronic liver disease (including Gilbert’s syndrome or asymptomatic gallstones) is acceptable if participant otherwise meets entry criteria.
15. Previous or concurrent malignancies other than multiple myeloma, unless the second malignancy has been considered medically stable for at least 2 years. The participant must not be receiving active therapy, other than hormonal therapy for this disease. Note: Participants with curatively treated non-melanoma skin cancer are allowed without a 2-year restriction.
16. Evidence of cardiovascular risk including any of the following:
    a. Evidence of current clinically significant untreated arrhythmias, including clinically significant ECG abnormalities including second degree (Mobitz Type II) or third-degree atrioventricular block.
    b. History of myocardial infarction, acute coronary syndromes (including unstable angina), coronary angioplasty, or stenting or bypass grafting within 3 months of screening.
    c. Class III or IV heart failure as defined by the New York Heart Association functional classification system.
    d. Uncontrolled hypertension.
17. Known immediate or delayed hypersensitivity reaction or idiosyncratic reaction to drugs chemically related to belantamab mafodotin, daratumumab, bortezomib, boron or mannitol or any other components of the study treatment.
18. Active infection requiring treatment.
19. Known HIV infection, unless the participant can meet all of the following criteria:
    a. Established anti-retroviral therapy for at least 4 weeks and HIV viral load <400 copies/mL
    b. CD4+ T-cell (CD4+) counts ≥350 cells/μL
    c. No history of acquired immunodeficiency syndrome-defining opportunistic infections within the last 12 months
20. Patients with hepatitis B will be excluded unless the following criteria can be met:

| Serology | Screening | During study treatment |
| --- | --- | --- |
| HBcAb+, HbsAg- | - Hepatitis B virus (HBV) DNA undetectable | - Monitoring per protocol - Antiviral treatment instituted if HBV DNA becomes detectable |
| HBsAg+ at screen  or within 3 months prior to first dose | - HBV DNA undetectable - Highly effective antiviral treatment started at least 4 weeks prior to first dose of study treatment - Baseline imaging per protocol - Participants with cirrhosis are excluded | - Antiviral treatment maintained throughout study treatment - Monitoring and management per protocol |

Note: presence of HBsAb indicating previous vaccination will not exclude a participant.

21. Positive hepatitis C antibody test result or positive hepatitis C RNA test result at screening or within 3 months prior to first dose of study treatment unless the participant can meet the following criteria:
    a. Negative RNA test.
    b. Successful anti-viral treatment (usually 8 weeks duration), followed by a negative hepatitis C virus RNA test after a washout period of at least 4 weeks.
22. Current corneal epithelial disease except for mild punctate keratopathy.
23. Intolerance or contraindications to anti-viral prophylaxis.
24. Symptomatic amyloidosis, active polyneuropathy, organomegaly, endocrinopathy, monoclonal plasma proliferative disorder, skin changes (POEMS) syndrome or active plasma cell leukemia at the time of screening.

**Supplementary Section 4: Ophthalmic assessments**

All patients were assessed by a qualified eye care specialist at baseline. Management of patients with corneal findings was performed in close communication with the GSK Medical Monitor and the coordinating eye care specialist.

Baseline ophthalmic examination for both eyes included:

1. Best corrected visual acuity.
2. Documentation of manifest refraction and the method used to obtain best corrected visual acuity.
3. Current glasses prescription (if applicable).
4. Anterior segment (slit lamp) examination with focus on the cornea and lens, including fluorescein staining of the cornea.
5. Intraocular pressure measurement.
6. Dilated funduscopic examination.

On-treatment and follow-up ophthalmic examination for both eyes included:

1. Best corrected visual acuity.
2. Documentation of manifest refraction and the method used to obtain best corrected visual acuity.
3. Anterior segment (slit lamp) examination with focus on the cornea and lens, including fluorescein staining of the cornea.
4. Intraocular pressure measurement (if clinically indicated).
5. Dilated funduscopic examination (if clinically indicated).

The end-of-treatment and last follow-up ophthalmic examination, if required, should match the baseline examination. Additional examinations were performed at the discretion of the treating eye care specialist.

**Supplementary Section 5: Figures**

**Figure S1. Study design of DREAMM-7**

^*^Reduce starting dose of dexamethasone to 10 mg for patients >75 years of age, who have a body-mass index <18.5 kg/m^2^, who had previous unacceptable adverse effects associated with glucocorticoid therapy, or who are unable to tolerate the starting dose.

AE, adverse event; BCMA, B-cell maturation antigen; BVd, belantamab mafodotin, bortezomib, and dexamethasone; DoR, duration of response; DVd, daratumumab, bortezomib, and dexamethasone; FPI, first patient in; IV, intravenous; LPI, last patient in; MM, multiple myeloma; MRD, minimal residual disease; OS, overall survival; PD, progressive disease; PFS, progression-free survival; q3w, every 3 weeks; q4w, every 4 weeks; q12w, every 12 weeks; qw, every week; R-ISS, Revised International Staging System; SC, subcutaneous.

**Figure S2. Swimmer plot of belantamab mafodotin treatment duration and response***

*Treatment duration is the time between the first dosing date and dosing end date, without accounting for dose interruptions.

CR, complete response; KVA, Keratopathy Visual Acuity; MR, minimal response; NE, not evaluable; PD, progressive disease; PR, partial response; sCR, stringent complete response; SD, stable disease; V, very good partial response.

**Supplementary Section 6: Tables**

**Table S1. Patient disposition**

|  | BVd  (N=10) | DVd (N=14) | Total (N=24) |
| --- | --- | --- | --- |
| **Patient status, n (%)** |  |  |  |
| Died | 0 | 4 (29) | 4 (17) |
| Ongoing | 9 (90) | 9 (64) | 18 (75) |
| On study treatment* | 4 (40) | 5 (36) | 9 (38) |
| In follow-up | 5 (50) | 4 (29) | 9 (38) |
| Withdrawn from study | 1 (10) | 1 (7) | 2 (8) |
| **Primary reason for withdrawal, n (%)^†^** |  |  |  |
| Lost to follow-up | 0 | 1 (7) | 1 (4) |
| Withdrawal by patient | 1 (10)^‡^ | 0 | 1 (4) |

*Any study treatment component. ^†^Patient could have only one primary reason for withdrawal. ^‡^Patient withdrew due to burden of procedure.

BVd, belantamab mafodotin, bortezomib, and dexamethasone; DVd, daratumumab, bortezomib, and dexamethasone.

**Table S2. Post-hoc efficacy results in patients with high-risk cytogenetic abnormalities including amp1q**

| Patients with high-risk cytogenetic abnormalities* | **BVd**  (N=7) | DVd (N=10) |
| --- | --- | --- |
| Experienced PFS event, n (%) | 3 (42.9) | 5 (50.0) |
| Complete response or better, n (%) | 2 (28.6) | 2 (20.0) |
| Very good partial response or better, n (%) | 5 (71.4) | 5 (50.0) |
| Partial response or better, n (%) | 6 (85.7) | 7 (70.0) |

*For this analysis, patients with high-risk cytogenetics had ≥1 of the following abnormalities: t(4;14), t(14;16), 17p13del, or amp1q.

BVd, belantamab mafodotin, bortezomib, and dexamethasone; DVd, daratumumab, bortezomib, and dexamethasone; PFS, progression-free survival.

**Table S3. Subsequent antimyeloma therapies**

|  | **BVd**  (N=10) | DVd (N=14) |
| --- | --- | --- |
| Received subsequent antimyeloma therapy, n (%) | 3 (30) | 8 (57) |
| Steroids | 3 (30) | 8 (57) |
| Immunomodulator | 1 (10) | 8 (57) |
| Monoclonal antibody | 2 (20) | 6 (43) |
| Proteasome inhibitor | 2 (20) | 5 (36) |
| Chemotherapy | 1 (10) | 5 (36) |
| Stem cell transplant | 1 (10) | 1 (7) |
| Engineered T/NK Cell Therapy | 0 | 1 (7) |
| Other* | 1 (10) | 0 |

*One patient in the BVd group who was classified under “Other” received daratumumab + vorhyaluronidase alfa in subsequent therapy.

**Table S4. TEAEs in ≥20% of patients in either treatment arm**

|  | BVd  (N=10) | DVd (N=14) |
| --- | --- | --- |
| Any TEAE, n (%)* | 10 (100) | 14 (100) |
| Thrombocytopenia | 7 (70) | 7 (50) |
| Foreign body sensation in eyes | 7 (70) | 0 |
| Blurred vision | 7 (70) | 0 |
| Diarrhea | 6 (60) | 2 (14) |
| Eye pain | 4 (40) | 0 |
| Photophobia | 4 (40) | 0 |
| Platelet count decreased | 3 (30) | 5 (36) |
| Malaise | 3 (30) | 3 (21) |
| Injection site erythema | 3 (30) | 2 (14) |
| Dry eye | 3 (30) | 1 (7) |
| Hepatic function abnormal | 3 (30) | 1 (7) |
| Blood lactase dehydrogenase increased | 3 (30) | 0 |
| Eye irritation | 3 (30) | 0 |
| Sensation of foreign body | 3 (30) | 0 |
| Constipation | 2 (20) | 3 (21) |
| Insomnia | 2 (20) | 3 (21) |
| Peripheral sensory neuropathy | 2 (20) | 3 (21) |
| Decreased appetite | 2 (20) | 2 (14) |
| Lymphopenia | 2 (20) | 1 (7) |
| Nausea | 2 (20) | 1 (7) |
| COVID-19 | 2 (20) | 0 |
| Neuropathy peripheral | 1 (10) | 4 (29) |
| White blood cell count decreased | 1 (10) | 4 (29) |
| Hypokalemia | 1 (10) | 3 (21) |
| Neutropenia | 1 (10) | 3 (21) |
| Hyperglycemia | 0 | 3 (21) |
| Leukopenia | 0 | 3 (21) |
| Muscle spasms | 0 | 3 (21) |

*TEAEs are presented by descending order by the number of patients in the BVd group.

BVd, belantamab mafodotin, bortezomib, and dexamethasone; DVd, daratumumab, bortezomib, and dexamethasone; TEAE, treatment-emergent adverse event.

**Table S5. AESIs by CTCAE**

|  | **BVd**  (N=10) | | DVd (N=14) | |
| --- | --- | --- | --- | --- |
|  | Any grade | Grade 3/4 | Any grade | Grade 3/4 |
| Any ocular AESI, n (%)* | 8 (80) | 0 | 1 (7) | 0 |
| Foreign-body sensation in eye | 7 (70) | 0 | 0 | 0 |
| Blurred vision | 7 (70) | 0 | 0 | 0 |
| Eye pain | 4 (40) | 0 | 0 | 0 |
| Photophobia | 4 (40) | 0 | 0 | 0 |
| Dry eye | 3 (30) | 0 | 1 (7) | 0 |
| Eye irritation | 3 (30) | 0 | 1 (7) | 0 |
| Sensation of foreign body | 3 (30) | 0 | 0 | 0 |
| Abnormal sensation in eye | 1 (10) | 0 | 0 | 0 |
| Conjunctival edema | 1 (10) | 0 | 0 | 0 |
| Conjunctivitis (allergic) | 1 (10) | 0 | 0 | 0 |
| Diplopia | 1 (10) | 0 | 0 | 0 |
| Lacrimation increased | 1 (10) | 0 | 0 | 0 |
| Any thrombocytopenic AESI, n (%)^†^ | 10 (100) | 10 (100) | 12 (86) | 10 (71) |
| Thrombocytopenia | 7 (70) | 7 (70) | 7 (50) | 7 (50) |
| Platelet count decreased | 3 (30) | 3 (30) | 5 (36) | 3 (21) |
| Any infusion-related reaction AESI, n (%)^‡^ | 1 (10) | 1 (10) | 3 (21) | 1 (7) |
| Diarrhea | 1 (10) | 1 (10) | 0 | 0 |
| Infusion-related reaction | 0 | 0 | 2 (14) | 0 |
| Rash | 0 | 0 | 1 (7) | 1 (7) |

*Ocular AEs are based on the MedDRA “corneal disorders” standardized MedDRA queries combined with preferred terms representing changes in visual acuity which were identified by GSK internal review. ^†^Thrombocytopenia is based on a hybrid of terms identified in the eCRF, and list of terms identified by GSK internal review. ^‡^Infusion-related reactions are based on a hybrid of terms identified in the eCRF, and list of terms identified by

GSK internal review. The event must start within 24 hours of infusion and lead to dose interruption/delay or study treatment withdrawal.

AE, adverse event; AESI, adverse event of special interest; BVd, belantamab mafodotin, bortezomib, and dexamethasone; CTCAE, Common Terminology Criteria for Adverse Events; DVd, daratumumab, bortezomib, and dexamethasone; eCRF, electronic case report form; MedDRA, Medical Dictionary for Regulatory Activities.

**Table S6. Ophthalmic examination findings (graded by the KVA scale)**

|  | BVd  (N=10) |
| --- | --- |
| Any ophthalmic examination findings, n (%)* | 10 (100) |
| Grade 3/4 events | 8 (80) |
| Events leading to dose reduction | 4 (40) |
| Events leading to dose interruption/delay | 10 (100) |

*Investigator-assessed ophthalmic examination findings (corneal examination findings and changes in best corrected visual acuity) graded by the KVA scale.

BVd, belantamab mafodotin, bortezomib, and dexamethasone; KVA, Keratopathy Visual Acuity.
